# Correction: Conditional generation of free radicals by selective activation of alkoxyamines: towards more effective and less toxic targeting of brain tumors

**DOI:** 10.1039/d6sc90135b

**Published:** 2026-06-22

**Authors:** Patricia Piris, Duje Buric, Toshihide Yamasaki, Paul Huchedé, Maïlys Rossi, Mélanie Matteudi, Marie-Pierre Montero, Anne Rodallec, Romain Appay, Christine Roux, Sébastien Combes, Eddy Pasquier, Marie Castets, Nicolas André, Paul Brémond, Manon Carré

**Affiliations:** a Centre de Recherche en Cancérologie de Marseille (CRCM), Inserm UMR1068, CNRS UMR7258, Aix-Marseille Université UM105, Institut Paoli Calmettes – Faculté de Pharmacie Marseille France manon.carre@univ-amu.fr; b Institut de Chimie Radicalaire, CNRS UMR7273, Aix-Marseille Université – Faculté des Sciences Marseille France; c Centre de Recherche en Cancérologie de Lyon (CRCL), Université Claude Bernard Lyon 1, INSERM 1052, CNRS 5286, Centre Léon Bérard Lyon France; d Service D'anatomie Pathologique et de Neuropathologie, Hôpital de La Timone, Assistance Publique-Hôpitaux de Marseille (APHM) Marseille France; e DOSynth Platform, Centre de Recherche en Cancérologie de Marseille (CRCM), Faculté de Pharmacie Marseille France; f Service D'Hématologie & Oncologie Pédiatrique, Hôpital de La Timone, Assistance Publique-Hôpitaux de Marseille (APHM) Marseille France

## Abstract

Correction for ‘Conditional generation of free radicals by selective activation of alkoxyamines: towards more effective and less toxic targeting of brain tumors’ by Patricia Piris *et al.*, *Chem. Sci.*, 2023, **14**, 7988–7998, https://doi.org/10.1039/D3SC01315D.

The authors regret that Philippe Mellet, Pierre Voisin, Sylvain Marque, and Gérard Audran were not included in the Acknowledgements of their published article. The corrected Acknowledgements section for this article is shown below.

**Acknowledgements**

We would like to thank S. Vigier for his help with animal experimentation, the Spectropole for NMR and HRMS analyses, N. Vanthuyne for chiral HPLC experiments, M. Giorgi for X-ray diffraction analyses, the ICEP platform of CRCM for their help in immunohistochemistry experiments, and Philippe Mellet, Pierre Voisin, Sylvain Marque, and Gérard Audran for fruitful discussions. This work was supported by research funding from charities (GEFLUC, RESOP, La Marie-Do, AROU, Association de Recherche contre le Cancer, Ligue Contre le Cancer) and institutions (Canceropôle PACA, Institut National du Cancer and Région Sud). This project has also received funding from the Excellence Initiative of Aix-Marseille University – A*MIDEX, a French “Investissements d'Avenir” program.

The Royal Society of Chemistry apologises for these errors and any consequent inconvenience to authors and readers.

